# Transglutaminase Activity Is Conserved in Stratified Epithelia and Skin Appendages of Mammals and Birds

**DOI:** 10.3390/ijms24032193

**Published:** 2023-01-22

**Authors:** Attila Placido Sachslehner, Marta Surbek, Bahar Golabi, Miriam Geiselhofer, Karin Jäger, Claudia Hess, Ulrike Kuchler, Reinhard Gruber, Leopold Eckhart

**Affiliations:** 1 Skin Biology Laboratory, Department of Dermatology, Medical University of Vienna, 1090 Vienna, Austria; 2 Clinic for Poultry and Fish Medicine, Department for Farm Animals and Veterinary Public Health, University of Veterinary Medicine Vienna, 1210 Vienna, Austria; 3 Department of Oral Biology, University Clinic of Dentistry, Medical University of Vienna, 1090 Vienna, Austria

**Keywords:** transglutaminase, cornification, keratinocytes, epidermis, filiform papillae, nail, feather, sweat gland, eccrine gland, gingiva, chicken, evolution, enzyme activity labeling

## Abstract

The cross-linking of structural proteins is critical for establishing the mechanical stability of the epithelial compartments of the skin and skin appendages. The introduction of isopeptide bonds between glutamine and lysine residues depends on catalysis by transglutaminases and represents the main protein cross-linking mechanism besides the formation of disulfide bonds. Here, we used a fluorescent labeling protocol to localize the activity of transglutaminases on thin sections of the integument and its appendages in mammals and birds. In human tissues, transglutaminase activity was detected in the granular layer of the epidermis, suprabasal layers of the gingival epithelium, the duct of sweat glands, hair follicles and the nail matrix. In the skin appendages of chickens, transglutaminase activity was present in the claw matrix, the feather follicle sheath, the feather sheath and in differentiating keratinocytes of feather barb ridges. During chicken embryogenesis, active transglutaminase was found in the cornifying epidermis, the periderm and the subperiderm. Transglutaminase activity was also detected in the filiform papillae on the tongue of mice and in conical papillae on the tongue of chickens. In summary, our study reveals that transglutaminase activities are widely distributed in integumentary structures and suggests that transglutamination contributes to the cornification of hard skin appendages such as nails and feathers.

## 1. Introduction

The skin of mammals, reptiles and birds functions as a barrier against the environment. In addition, skin appendages, such as hair, nails, scales, and feathers play critical roles in thermoregulation, locomotion, defense and attack [1,2]. The surface of the skin and the main portion of skin appendages are formed by epithelial cells known as keratinocytes. Maintenance of stem cells and proliferation of keratinocytes drives the regeneration and growth of epithelial structures, whereas cell differentiation and cornification establish the high mechanical resistance which is critical for the function of most epithelial structures. By cornification, living keratinocytes are converted into dead cell remnants which remain connected to each other and to the living epithelium [3,4,5]. Thereby, cornified keratinocytes, also known as corneocytes, become the building blocks of macroscopic structures, such as the cornified surface of the epidermis, hair and nails.

Cornification depends on intracellular protein cross-linking which is achieved by two mechanisms, i.e., transglutamination and disulfide bonding. Transglutamination refers to the enzymatically catalyzed formation of an isopeptide bond between a glutamine and a lysine residue, whereas disulfide bonds form between two cysteine residues under oxidizing conditions and upon catalysis by sulfhydryl oxidases [5,6,7]. Hard skin appendages such as hair, feathers, nails and claws contain high amounts of cysteine-rich proteins which are cross-linked by disulfide bridges. By contrast, the keratinocytes of the epidermis and the hair root sheath do not contain these structural proteins and largely rely on transglutamination for cornification [3,4]. Consequently, the enzymes that catalyze transglutamination, i.e., transglutaminases (TGMs) are essential for the proper formation of the epidermal stratum corneum, the hair cuticle and the inner root sheath of hair follicles. Mutations of TGM1, TGM3 and TGM5 cause the human diseases lamellar ichthyosis, uncombable hair syndrome and acral peeling skin syndrome, respectively [8,9,10]. Furthermore, TGM activity is high in the stratified epithelium of the esophagus [11]. However, it has remained unknown whether the activity of TGMs is also involved in the differentiation of other epithelia and skin appendages in mammals. In birds, the epidermis of the skin and scutate scales as well as the epithelium of the esophagus contain TGM activity [11], whereas TGM activity in other skin appendages has not yet been reported.

Here, we investigated a selection of stratified epithelia and integumentary appendages of mammals and birds for TGM activity. We show that epithelial TGM activity is more widely distributed than previously thought, including hard skin appendages and cornified segments of skin glands.

## 2. Results

### 2.1. Fluorescence Labeling Detects TGM Activity in Terminally Differentiated Keratinocytes of the Epidermis and Oral Epithelium

The aim of this study was to compare stratified epithelia and epithelial appendages of amniotes with regard to the presence and intra-epithelial localization of TGM activity. To this end, we prepared cyrosections of tissue samples and incubated them with the universal TGM substrate cadaverine linked with the Alexa-555 fluorophore. In the presence of 5 mM Ca^2+^ ions, TGMs present in the tissue section catalyze the isopeptide bond formation between an amino group of Alexa-555-cadaverine and the γ-carboxamide group of glutamine residues of proteins on the section. Negative control reactions were performed by omitting exogenous Ca^2+^ and blocking endogenous Ca^2+^ with EDTA. The assay of TGM activity is based on protocols which have been validated using samples from normal and TGM1-deficient human skin. Tissues lacking endogenous TGM1 do not show signals in the in situ TGM activity assay [12,13].

**Figure 1:**
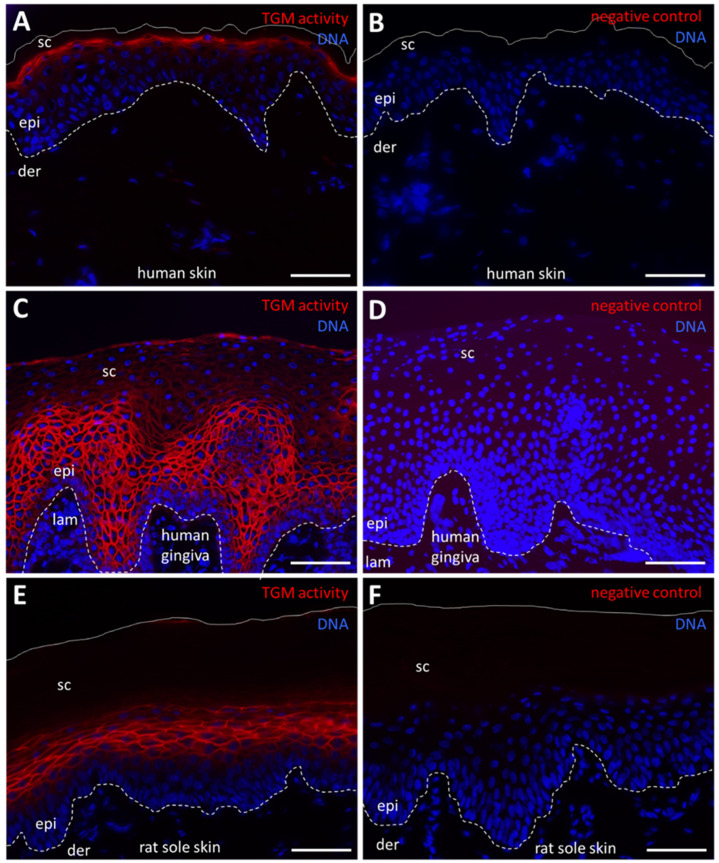
Transglutaminase (TGM) activity in the epidermis and gingival epithelium. (**A**) TGM activity (red) in the human epidermis. A membrane-associated signal was detected in the outermost nucleated cell layer of the epidermis. (**B**) In negative control experiments, CaCl_2_ was replaced with 5 mM EDTA. (**C**) TGM activity in the human gingiva. A membrane-associated signal was detected in several layers above the basal layer and in the outmost layer. (**D**) In negative control experiments, CaCl_2_ was replaced with 5 mM EDTA. (**E**) TGM activity in the sole skin of a rat. (**F**) In negative control experiments, CaCl_2_ was replaced with 5 mM EDTA. Dashed lines indicate the basement membrane. Continuous lines indicate the surface of the skin. der, dermis; epi, epidermis; lam, lamina propria; sc, stratum corneum. Scale bars: 50 µm (**A**,**B**,**E**,**F**), 100 µm (**C**,**D**).

To confirm the specificity of the assay, we stained skin and oral mucosa, which are known as sites of TGM1 expression [14,15]. TGM activity was detected in the granular layer of human epidermis (Figure 1A), suprabasal cells of human gingival epithelium (Figure 1C) and suprabasal cells of the epidermis of rat sole skin (Figure 1E). As hair follicles have also been reported to contain active TGM, we labeled sections through the mouse snout skin, which contains large hair follicles. TGM activity was detected at high intensity in the inner root sheath and cuticle and at low levels in the hair cortex (Supplementary Figure S1).

### 2.2. Cornifying Papillae of the Tongues of Mammals and Birds Contain Active Transglutaminases

**Figure 2:**
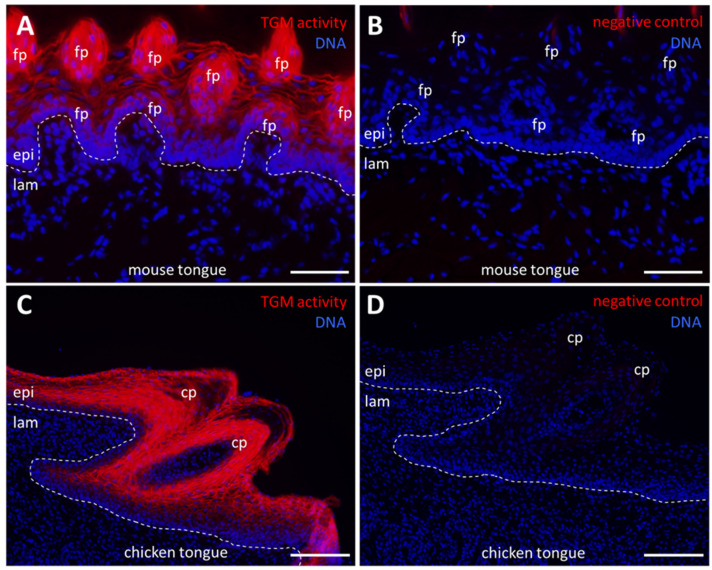
Transglutaminase (TGM) activity in the tongue of mouse and chicken. (**A**) TGM activity (red) in the mouse tongue epithelium and in the filiform papillae. The section is slightly oblique. (**B**) Negative control in which CaCl_2_ was replaced by 5 mM EDTA. (**C**) TGM activity in an embryonic chicken tongue epithelium and in the conical papillae. (**D**) Negative control in which CaCl_2_ was replaced by 5 mM EDTA. Dashed lines indicate the basement membrane. cp, conical papilla; epi, epithelium; lam, lamina propria; fp, filiform papilla. Scale bars: 50 µm (**A**,**B**), 100 µm (**C**,**D**).

The tongue of mammals contains fungiform papillae, where taste buds are located, and filiform papillae, which are involved in the sensation of touch and in the mechanical interaction with food. Filiform papillae are cone-shaped and their epithelium is cornified. In birds, conical papillae are present at the proximal end of the tongue. We investigated tongues of mice and chickens. In mice, TGM activity was readily detectable in suprabasal cells of the epithelium but not in the mesenchymal lamina propria (Figure 2A,B). Similarly, the epithelial cells of the filiform papillae contained TGM activity (Figure 2A,B). The activity was localized to the periphery of cells, suggesting that it was caused by membrane-anchored TGM1. In the chicken, TGM activity was detected in suprabasal epithelial cells of conical papillae and in the mucosal epithelium (Figure 2C,D).

### 2.3. TGM Activity Is Present in the Nail Matrix

**Figure 3:**
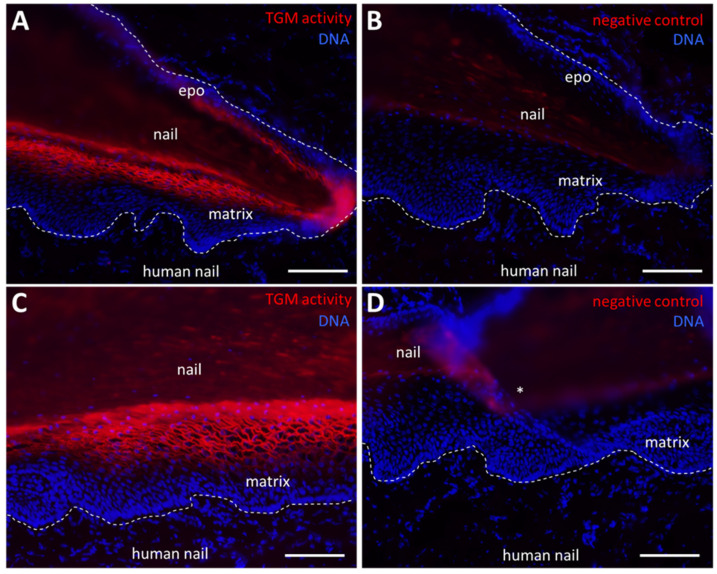
Transglutaminase (TGM) activity in the human nail matrix. (**A**) TGM activity (red) in the human nail. The nail matrix shows TGM activity and a membrane associated signal. (**B**,**D**) In negative control experiments CaCl_2_ was replaced by 5 mM EDTA. (**C**) Detail of the human nail matrix. Dashed lines indicate the basement membrane. epo, eponychium. An asterisk indicates a fold in the section due to a preparation artifact. Scale bars: 100 µm (**A**,**B**), 50 µm (**C**,**D**).

Next we investigated cryopreserved samples of the human nail apparatus [16]. TGM activity was detected in a membrane-associated pattern in the suprabasal keratinocytes of the nail matrix, whereas basal cells of the nail matrix were negative for TGM activity labeling (Figure 3A,C). Replacement of CaCl_2_ with EDTA abolished the staining, confirming that a calcium-dependent enzyme is required for the attachment of the fluorophore-tagged cadaverine (Figure 3B,D). In support of the homology of mammalian nails and avian claws, we detected TGM activity also in the matrix of chicken claws (Supplementary Figure S2).

### 2.4. TGM Activity Contributes to Formation of the Arcosyringeal Duct of Sweat Glands

**Figure 4:**
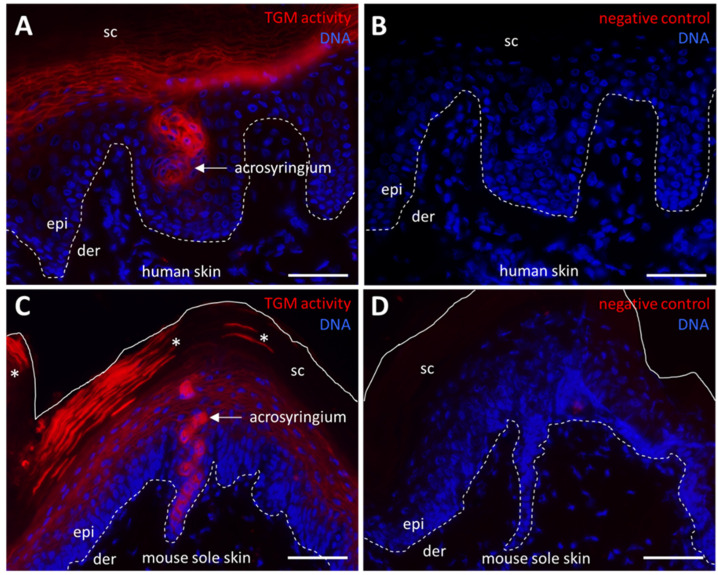
Transglutaminase (TGM) activity in the sweat gland duct. (**A**) TGM activity in the arcosyringium of a sweat gland in human skin. (**B**) Negative control in which CaCl_2_ was replaced by 5 mM EDTA. (**C**) TGM activity in the arcosyringium of a sweat gland in mouse sole skin. Asterisks indicate TGM activity present in corneocytes of the stratum corneum (sc). (**D**) Negative control in which CaCl_2_ was replaced by 5 mM EDTA. Der, dermis; epi, epidermis; sc, stratum corneum. Scale bars: 50 µm.

We next expanded the analysis of TGM activity to eccrine sweat glands which develop, by invagination of the epidermis and keratinocyte differentiation, into secretory cells and duct cells (Supplementary Figure S3). Sweat is secreted in the deepest portion of the gland from where it passes through the duct to the surface of the epidermis. Fluorophore–cadaverine labeling showed that the acrosyringium, that is, the segment of the duct which passes through the epidermis, contains active TGM both in human (Figure 4A, Supplementary Figure S3) and mouse (Figure 4C) sweat glands. Negative controls confirmed the specificity of the enzyme-mediated labeling (Figure 4B,D). Interestingly, we also observed a consistent labeling of corneocytes in the vicinity of sweat gland openings in mouse footpad skin (Figure 4C, asterisks), indicating that TGM was retained in an active state in these cornified keratinocytes whereas no such activity was detected by our assay in other parts of murine stratum corneum and in human stratum corneum.

### 2.5. TGM Activity Is Present in Differentiated Keratinocytes of the Feather Follicle

**Figure 5:**
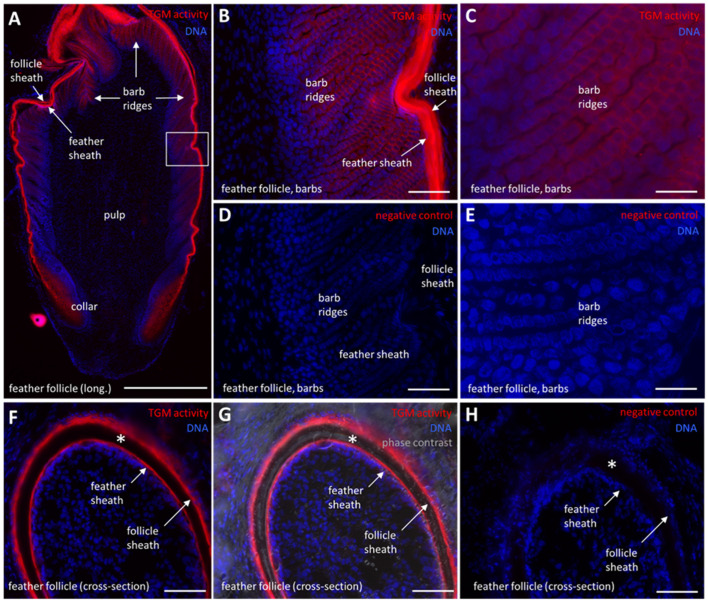
Transglutaminase (TGM) activity in feather follicles. (**A**) TGM activity (red) in a longitudinal (long.) section through a feather follicle. The white box indicates the area depicted at larger magnifications in panels (**B**,**C**). A black asterisk indicates an unspecifically labeled spot on the slide. (**B**,**C**) TGM activity in the developing feather barbules. (**D**,**E**) Negative control in which CaCl_2_ was replaced by 5 mM EDTA. (**F**) TGM activity in a cross-section of a feather follicle and the surrounding follicle sheath. (**G**) TGM activity in a cross-section of a feather follicle, merged with differential interference (phase) contrast. The mature cornified epithelium (white asterisk) lacks TGM activity. (**H**) Negative control to panel (**F**) in which CaCl_2_ was replaced by 5 mM EDTA. Scale bars: 500 µm (**A**), 50 µm (**B**,**D**,**F**–**H**), 20 µm (**C**,**E**).

In a previous study, we detected TGM activity in a membrane-associated pattern in the epidermis and scutate scales of chickens [11]. Here, we show that TGM activity is also present in scutate scales during late embryonic development (Supplementary Figure S4). Interestingly, the periderm and subperiderm, which are embryo-specific superficial layers of the epithelium, contain active TGM (Supplementary Figure S4). Next, we investigated feather follicles, which have not been studied previously with regard to transglutamination [11]. TGM activity was detected in the follicle sheath (Figure 5A,F,G), which is an epithelium continuous with the epidermis. Furthermore, strong TGM activity was detected in the feather sheath (Figure 5A,B,F,G) which envelopes the growing feather before it disintegrates to allow the emergence of the feather. Most interestingly, TGM activity was also present, though at lower levels, in the barb ridges (Figure 5A–C), where keratinocytes undergo cornification to build the barbules of mature feathers. The pulp of the feather follicle and keratinocytes in the basal layers of feather follicle epithelia lacked TGM activity (Figure 5A). Negative controls confirmed that that labeling was mediated by a calcium-dependent enzyme (Figure 5D,E,H).

## 3. Discussion

The results of this study demonstrate that TGM activities are present in a broad range of integumentary structures in amniotes. Consistently, the TGM activity was localized in differentiated epithelial cells within which it was predominantly associated with the cell membrane, although not in all skin appendages. Thus, transglutamination is a type of protein cross-linking which has been conserved in many pathways of keratinocyte differentiation during the diversifying evolution of fully terrestrial vertebrates.

In line with previous reports [17], our investigation revealed expression and catalytic activity of TGMs in the epidermal and oral epithelia. These stratified epithelia are generally similar with regard to the keratinocyte differentiation program whereby parts of the oral epithelium do not undergo cornification [18]. Our data revealed that the human gingival epithelium contains active TGM, suggesting that this part of the oral epithelium, which is exposed to high levels of mechanical stress, is strengthened by protein cross-linking. Likewise, transglutamination appears to protect the epithelium on the surface of the tongue and in lingual papillae. Recently, immunolabeling studies using cross-reactive antibodies have suggested expression of TGMs in suprabasal epithelial cells of avian tongue [19]. The localization of TGM enzymatic activity in our study is congruent with the pattern of immunoreactivity [19].

The detection of TGM activity in filiform papillae of the tongue is interesting because these cornified papillae resemble hair and nail with regard to the expression of cysteine-rich keratins [20,21], presence of trichohyalin-rich epithelial cells [22] and nuclear DNA degradation by DNase1L2 [23], suggesting that they have evolved from a common evolutionary precursor. In support of the shared role of TGM activity in these integumentary appendages, we can demonstrate that the human nail matrix (Figure 3) and the chicken claw matrix (Supplementary Figure S2) contain abundant TGM activity. Furthermore, mutations in *TGM1* caused ultrastructural defects of nail corneocytes [24,25] and substantial isopeptide cross-linking was detected in hair [26]. Together, the localization of TGM activity in differentiating epithelial cells and the detection of transglutaminated protein structures suggests that protein transglutamination is conserved in nail, hair and filiform papillae of the tongue.

Feathers are complex hard skin appendages exclusively found in birds [27,28]. The evolutionary history of feathers is still a matter of scientific debate [29,30,31,32], but several lines of evidence point to a convergent evolution of cysteine-rich protein components [33,34] and to an origin of feathers that is independent from nails and hair [31]. Our results show that TGM activity is particularly prominent in the feather sheath which has a similar function as the inner root sheath of hair. Both support the growth of the respective skin appendage without becoming a part of the mature structure. In addition, TGM activity is present in barb ridge keratinocytes which form the feather barbs. However, the low staining levels suggest that this activity is less critical, possibly resembling TGM activity within the hair shaft [26]. In support of a role of isopeptide bonds in feathers, DTT-insoluble cornified envelopes were detected in barbs, barbules and the rachis of feathers [26]. One hypothesis about the evolution of feathers holds that the feather sheath is derived from the embryonic periderm while the cornified parts of the feather are derived from the subperiderm of embryonic skin [30,33]. Here, we show that both periderm and subperiderm contain TGM activity, suggesting that TGM activity in these embryonic epithelial layers and in feathers may have a common evolutionary origin.

In sweat glands, TGM activity is restricted to the acrosyringium, whereas the secretory portion of these glands lacks significant TGM activity. This pattern aligns well with the expression pattern of TGM substrates such as involucrin, loricrin and filaggrin [35,36,37]. We propose that transglutamination-mediated crosslinking of proteins stabilizes the acrosyringium within the highly dynamic environment of the epidermis, in which keratinocytes continuously proliferate in the basal layer and move through the suprabasal layers, cornify, and eventually are shed by desquamation. The sweat gland duct needs to withstand the changes of mechanical forces in this epithelium in order to ensure a constantly open path for sweat secretion. Obstruction of sweat glands and hypohidrosis was reported for patients carrying mutations of *TGM1* [38]. Considering the recent advances in the understanding, diagnosis and therapy of TGM-dependent diseases [39,40,41,42,43,44,45,46], it will be interesting to further explore the roles of TGMs in sweat glands and other skin appendages.

These results have been obtained in a comparative study of tissues using the same assay, in situ labeling of TGM activity, for all samples. Compared to immunolabeling protocols, this assay has the advantage that it does not depend on the conservation of antibody epitopes across TGM homologs. In contrast to the detection of TGM proteins by immunolabeling, our assay informs not only about expression but also about the activation status of TGM. This is important because post-translational modifications have been suggested to alter the activity of TGM1 and TGM3 by at least one order of magnitude [47,48]. However, the present study also has important limitations. First, the molecular identity of the active TGMs remains to be determined. A cell membrane-associated pattern of activity suggests that it is caused by TGM1, the only TGM containing a membrane anchor. Further studies using mRNA in situ hybridization or immunostaining with specific antibodies are required. Another limitation of our study is that the conditions, such as cytoplasmic calcium concentrations, change significantly within tissues in vivo [49] whereas optimal conditions are provided by the assay buffer across the entire tissue section. Therefore, some tissue sites may contain less TGM activity in vivo than in vitro.

We conclude that in situ labeling of TGM activities is a valuable approach for the comparative analysis of cornification in the integument of mammals and birds. Future studies will be aimed at dissecting the contributions of individual TGM enzymes to cornification of stratified epithelia and skin appendages in terrestrial vertebrates.

## 4. Materials and Methods

### 4.1. Tissue Samples

Human skin was prepared from tissue excised during plastic surgery procedures. The study was conducted according to the guidelines of the Declaration of Helsinki and approved by the Ethics Committee of the Medical University of Vienna (approval EK. 1969/2021, date of approval 18 November 2021). Human toe samples were obtained from male individuals who had died of diseases that are not associated with skin abnormalities or nail dystrophies. The study was conducted according to the guidelines of the Declaration of Helsinki and approved by the Ethics Committee of the Medical University of Vienna (approval EK. Nr. 131/2004, date of approval 19 April 2004) [16]. Human gingiva was prepared in the course of tooth extractions. The study was conducted according to the guidelines of the Declaration of Helsinki and approved by the Ethics Committee of the Medical University of Vienna (approval EK Nr. 631/2007, date of approval 23 January 2008) [50]. Informed consent was obtained from all subjects involved in the study.

Tissue samples were prepared from mice (strain C57BL/6J) and rats (strain Sprague Dawley, 10 weeks old) immediately after killing the animals. The Ethics Committee of the Medical University of Vienna decided that, in agreement with the national laws of Austria, a permission for killing mice and rats for organ preparation was not required.

For the study of avian epithelial cornification, tissue samples were prepared from commercial broiler chickens (Ross-308, 21 days old) and chicken embryos from eggs at day 18 day of incubation, corresponding to Hamburger and Hamilton stage 44 [51], VALO Biomedica, Osterholz-Scharmbeck, Germany. Samples were obtained from untreated chickens which were maintained in a trial approved by the ethics and animal welfare committee of University of Veterinary Medicine, Vienna, Austria and the Austrian Federal Ministry of Education, Science and Research (license number BMBWF GZ: 2021-0.881.824).

### 4.2. TGM Activity Labeling In Situ

Tissue samples were dissected freshly and immediately washed in phosphate-buffered saline (PBS) before they were embedded in optimal cutting temperature (OCT) compound (Scigen, Paramount, Canada) and further snap-frozen in liquid nitrogen. Feather follicles were isolated according to a published protocol [52]. Cryosections of 6 µm thickness were cut with a cryostat (Leica CM3050S) and either stored at −80 °C or stained immediately. The in situ TGM activity assay was performed according to published protocols [11,12,13,53] with modifications. Briefly, cryosections were placed in PBS for 5 min at room temperature to wash off the OCT compound from the slides. The sections were incubated with 1% bovine serum albumin in 0.1 M Tris-HCl, pH 7.4 at room temperature for 30 min before incubation with 5 µM Alexa-fluor-555-cadaverine (Thermo Fisher Scientific, Waltham, MA, USA) in 0.1 M Tris-HCl pH 7.4 and 5 mM CaCl_2_ for 2 h at room temperature in a dark chamber. In negative control experiments, CaCl_2_ was replaced by 5 mM EDTA. The TGM reaction was stopped by application of 25 mM EDTA in PBS for 5 min, which was followed by an incubation with 1 µg/mL Hoechst 33258 (Molecular Probes, Eugene, OR, USA) at room temperature. Finally, the sections were mounted with Permafluor (Thermo Fisher Scientific, Waltham, MA, USA). Samples were examined with an Olympus BX63 microscope and images were obtained with an Olympus UC-90 camera. Fluorescent images and images obtained under phase contrast were merged with cellSens Dimensions (version 1.16).

## Data Availability

Data is contained within the article or Supplementary Materials.

## Supplementary Materials

The following are available online at https://www.mdpi.com/article/10.3390/ijms24032193/s1.
