# Supplementary material for: Transglutaminase Activity Is Conserved in Stratified Epithelia and Skin Appendages of Mammals and Birds
Source: Int J Mol Sci. 2023 Jan 22;24(3):2193. doi: 10.3390/ijms24032193 (PMC9916842; doi:10.3390/ijms24032193)
Supplement: Supplementary file 1 [file ijms-24-02193-s001.zip › ijms-2102212-supplementary.pdf]

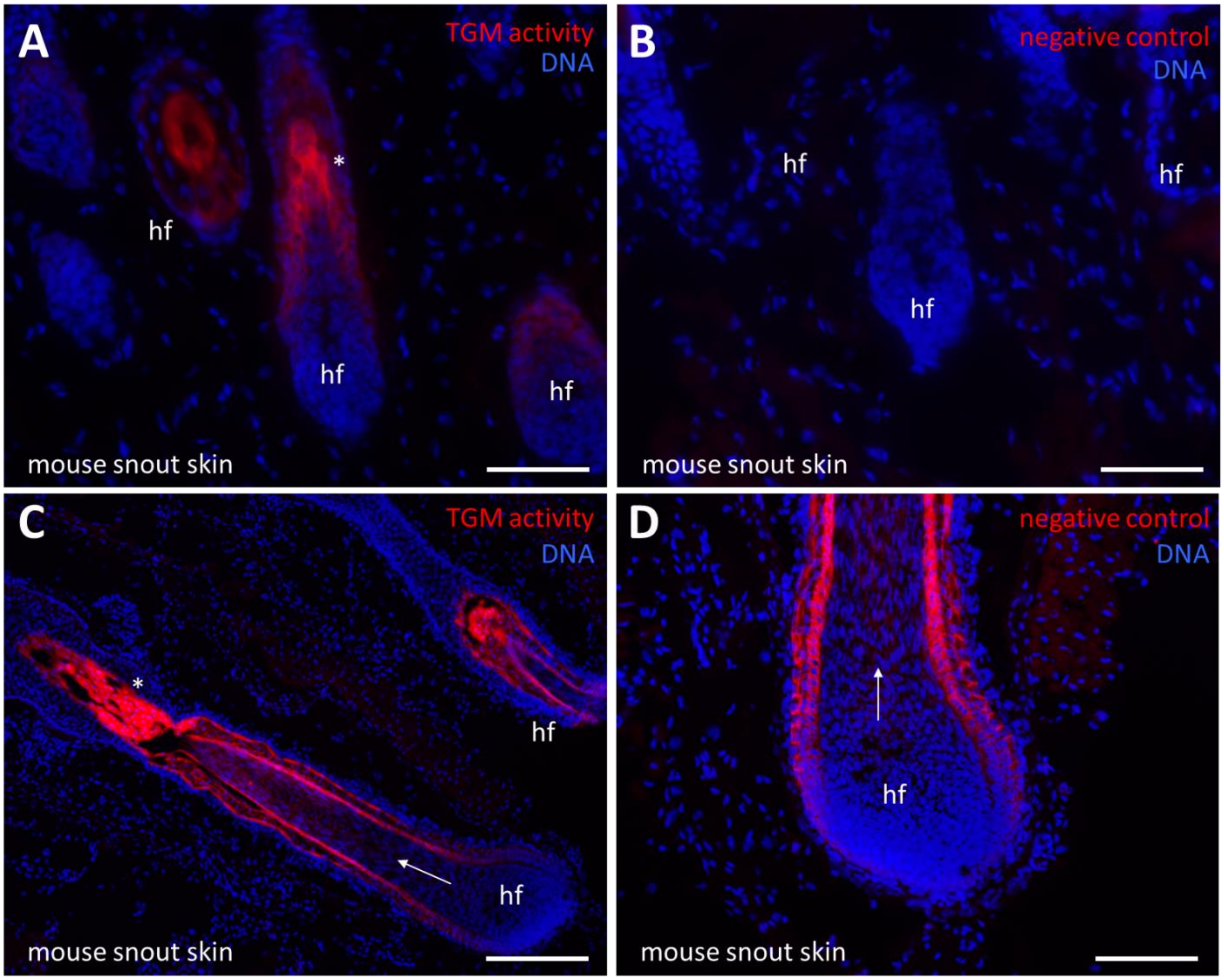

Figure S1. Transglutaminase (TGM) activity in the murine hair follicles. Mouse snout skin containing hair follicles (hf) was subjected to cryosectioning. The sections were incubated with Alexa 555-cadaverine in the presence of calcium ions to label TGM activity (red) (**A**, **C, D**). In negative control experiments, $CaCl_2$ was replaced with 5 mM EDTA (**B**). Note that, apart from one cross-section in the upper part of panel **A**, sections are oblique relative to the main growth axis of the hair shafts. Panels **C** and **D** show large follicles from which whiskers grow. The direction of growth is indicated by arrows. Asterisks indicate parts of the hair root sheath, which are tangentially cut through the TGM activity-positive layers (**A**, **C**). Scale bars: 50 µm (**A**, **B**, **D**), 100 µm (**C**).

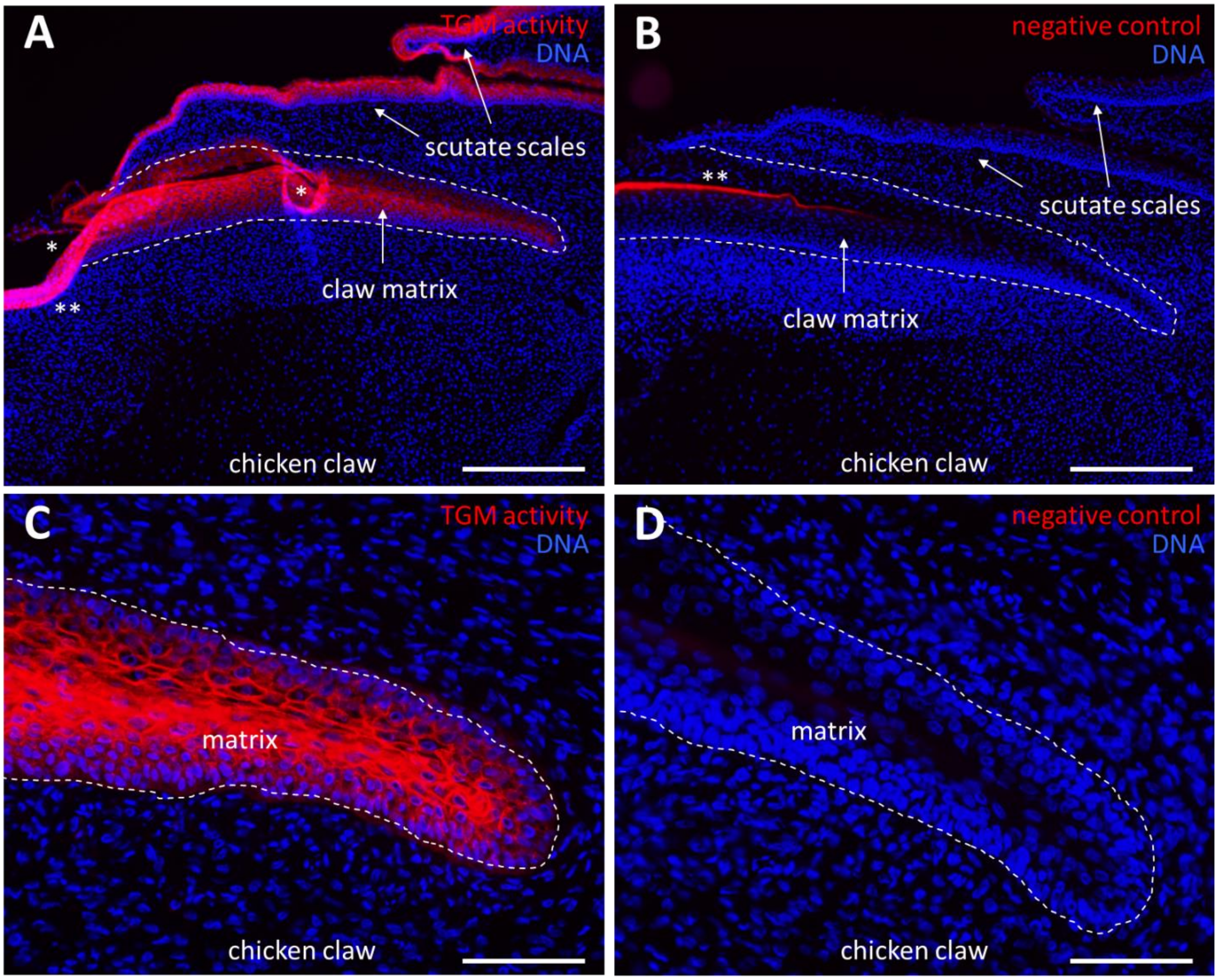

Figure S2. Transglutaminase (TGM) activity in the chicken claw matrix. TGM activity was labeled (red) in the claw matrix at stage HH 44 of chicken embryonic development (**A**, **C**). In negative control experiments, $CaCl_2$ was replaced with 5 mM EDTA (**B**, **D**). The folding of parts of the cornified claw is an artifact of sectioning (single asterisks). Double asterisks indicate an autofluorescence signal in the cornified part of the claw. Dashed lines indicate the basement membrane around the claw matrix. Scale bars: 200 µm (**A**, **B**), 50 µm (**C**, **D**).

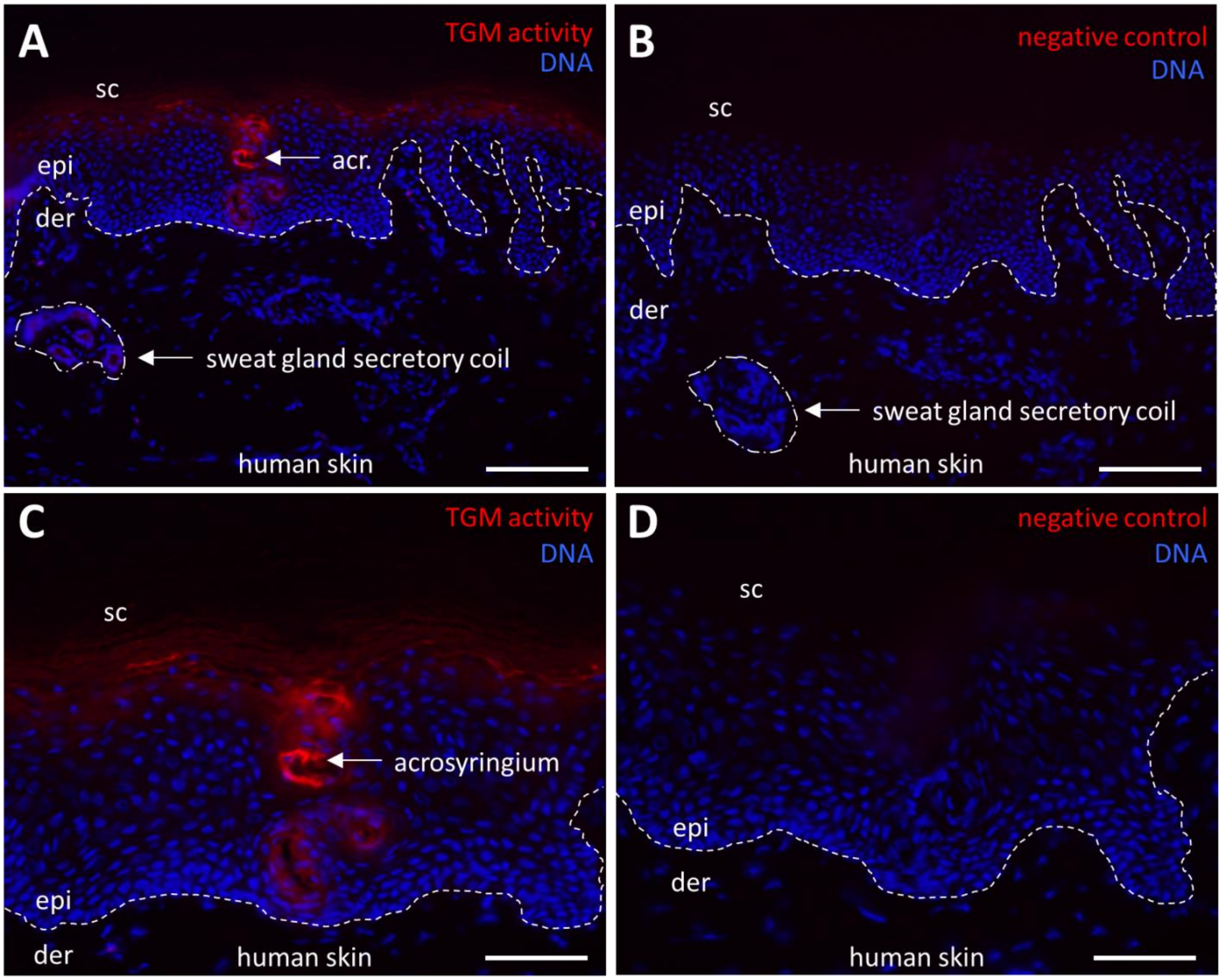

Figure S3. Transglutaminase (TGM) activity in human sweat glands. TGM activity was localized by *in situ* fluorescence labeling (red) in human skin sections containing sweat glands (**A**, **C**). In negative control experiments $CaCl_2$ was replaced by 5 mM EDTA (**B**, **D**). acr, acrosyringium; der, dermis; epi, epidermis. Scale bars: 100 µm (**A**, **B**), 50 µm (**C**, **D**).

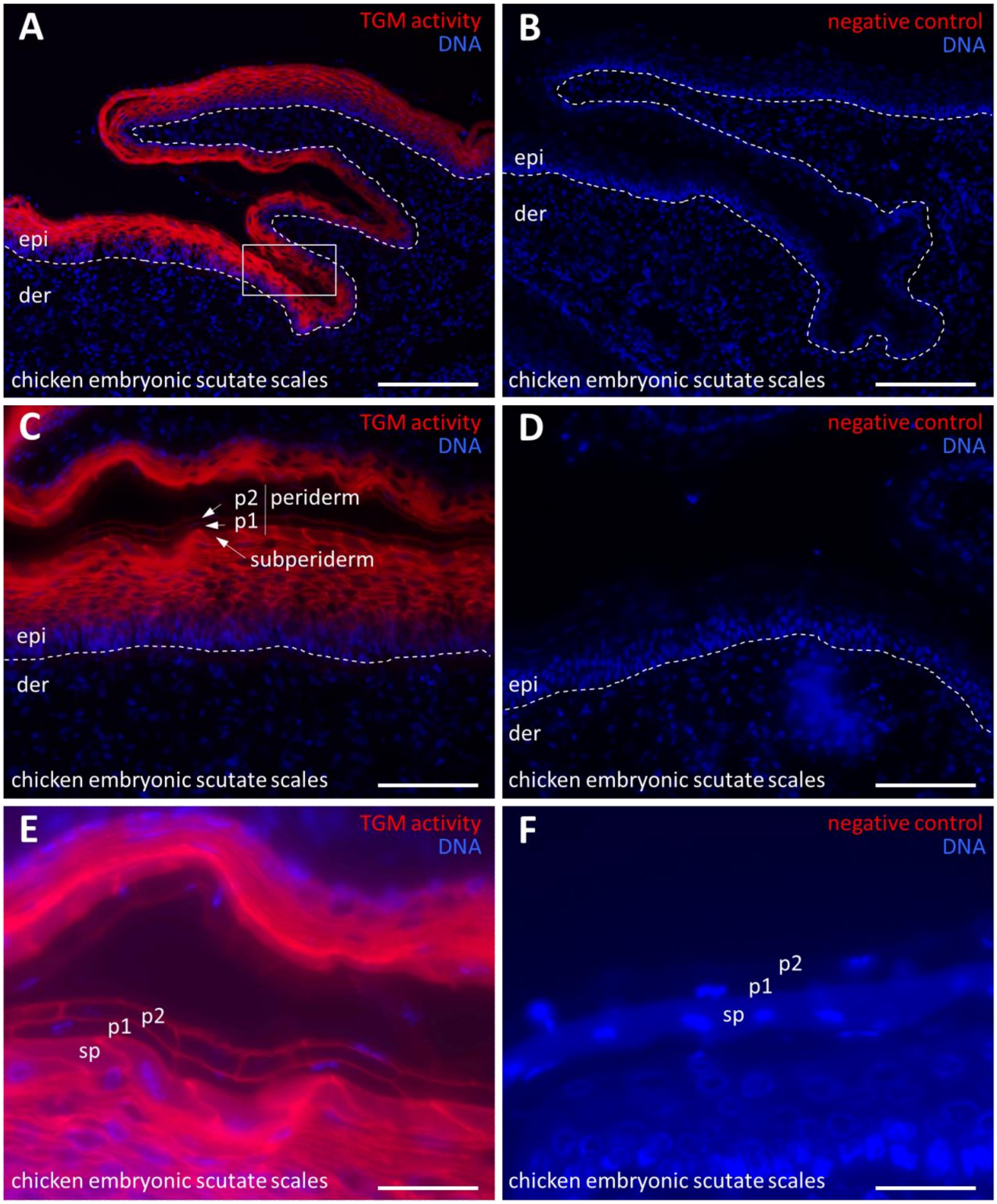

Figure S4. Transglutaminase (TGM) activity in the avian periderm and subperiderm. TGM activity was labeled (red) in scutate scales at stage HH44 of chicken embryonic development (**A**, **C**, **E**). In negative control experiments, $CaCl_2$ was replaced with 5 mM EDTA (**B**, **D**, **F**). Dashed lines indicate the dermo-epidermal junction. der, dermis; epi, epidermis; p1, first periderm layer; p2, second periderm layer; sp, subperiderm. The brightness of the images in **E** and **F** was increased by 50%. Scale bars: 100 µm (**A**, **B**), 50 µm (**C**, **D**), 20 µm (**E**, **F**).
